# The fracture strength by a torsion test at the implant-abutment interface

**DOI:** 10.1186/s40729-015-0027-x

**Published:** 2015-10-10

**Authors:** Fumihiko Watanabe, Kazuhiko Hiroyasu, Kazuhiko Ueda

**Affiliations:** 1 Department of Crown and Bridge Prosthodontics, School of Life Dentistry at Niigata, The Nippon Dental University, 951-8580 1-8 Hamaura-cho, Chuo-ku, Niigata Japan; 2 Oral Implant Care Unit Niigata Hospital, The Nippon Dental University, 1-8 Hamaura-cho, Chuo-ku, Niigata Japan

**Keywords:** Torsion test, Implant-abutment interface, Abutment connection deformation, Torque, Mechanical load

## Abstract

**Background:**

Fractured connections between implants and implant abutments or abutment screws are frequently encountered in a clinical setting. The purpose of this study was to investigate fracture strength using a torsion test at the interface between the implant and the abutment.

**Methods:**

Thirty screw-type implant with diameters of 3.3, 3.8, 4.3, 5.0, and 6.0 mm were submitted to a torsion test. Implants of each size were connected to abutments with abutment screws tightened to 20 N · cm. Mechanical stress was applied with a rotational speed of 3.6 °/min until fracture occurred, and maximum torque (fracture torque) and torsional yield strength were measured. The mean values were calculated and then compared using Tukey’s test. The abutments were then removed, and the implant-abutment interfaces were examined using a scanning electron microscope (SEM).

**Result:**

No significant differences in mean fracture torque were found among 3.3, 3.8, and 4.3 mm-diameter implants, but significant differences were found between these sizes and 5.0 and 6.0 mm-diameter implants (*p* < 0.01). Concerning mean torsional yield strength, significant differences were found between 3.3, 3.8, and 4.3 mm-diameter and 5.0 and 6.0 mm-diameter implants (*p* < 0.01). Observations under the SEM showed that all the projections of the abutment corresponding to the internal notches of the implant body had been destroyed.

**Conclusions:**

Smaller diameter implants demonstrated lower fracture torque and torsional yield strength than implants with larger diameters. In internal tube-in-tube connections, three abutment projections corresponding to rotation-prevention notches were destroyed in each implant.

## Background

A single-tooth osseointegrated implant is composed of an implant body, an abutment, an abutment screw, and an artificial crown. The implant and abutment are typically connected by an abutment screw. The degree of mechanical integrity at the implant-abutment interface depends on screw preload, abutment connection design, the fitness of all components, and dynamic loading conditions. Fractures of the internal or external connection between implants and implant abutments or abutment screws are encountered in the clinical setting [1–3]. Fractures may occur as a result of excessive axial forces (bending moments), horizontal loading, or rotational torque during functional activities such as chewing or parafunctional activities such as grinding and clenching. Several studies [4, 5] have evaluated implant-abutment assemblies in dynamic cyclic fatigue testing according to ISO 14801 [6]. However, evaluation of the strength of the implant-abutment assembly has not been reported when subjected to torsion testing. Test methods and standard vales have not yet been established for such torsion testing of implant-abutment assemblies. Abutment connections are principally classified as either internal or external types and are available in a variety of designs, including hexagonal, octagonal, cone screw, cone hex, cylinder hex, spline, cam, cam tube, and pin/slot. Implants incorporate features for rotation-prevention at the implant-abutment interface. The advantages and disadvantages of various types of implant-abutment connections have been discussed in several studies [7–11]. Norton reported that internal implant-abutment interface connections have higher bending moment resistance than external connections [10]. Presently, although more than 200 kinds of implant systems are available on the market, most manufacturers do not provide specific data regarding system-specific implant-abutment connection design complications [12, 13]. In the oral cavity, implant restorations are exposed to vertical, horizontal, and rotational forces during chewing. The maximum torsional strength and proportional limited strength on crown and tooth restoration materials were measured, and the values were compared with a mechanical bending and pull test value [14].

The purpose of this in vitro study was to investigate fracture strength using a torsion test at the implant-abutment interface in order to assess the effect of torsion force on the connection.

## Methods

**Fig. 1:**
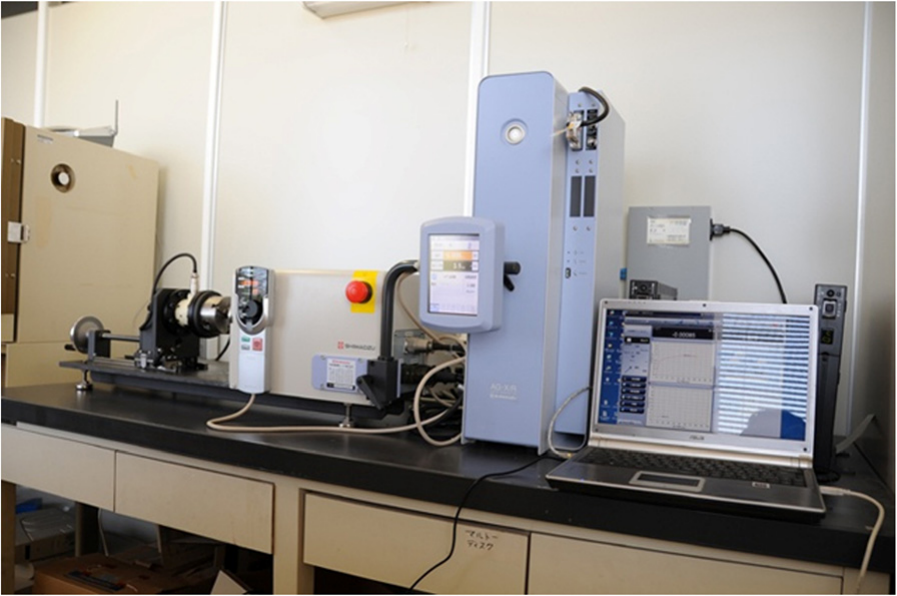
Torsion testing device

Thirty titanium screw implants of five different diameters (3.3, 3.8, 4.3, 5.0, and 6.0 mm) with tube-in-tube implant-abutment connections (CAMLOG Biotechnologies, Wimsheim, Germany) were used as test specimens. Implants of each size were connected to abutments, and abutment screws were tightened to 20 N · cm using a digital torque meter (Hios HDM-5; Hios Inc, Chiba, Japan). A torsion testing device (AG-XR; Shimadzu, Kyoto, Japan) was used on all implant test specimens in this study (Fig. 1).

The specimens were mounted on the torsion testing device, and mechanical stress was applied with a rotational speed of 3.6 °/min until permanent deformation or fracture occurred. The maximum torque (fracture torque) and torsional yield strength were measured in each specimen. The mean values of measured data were calculated and compared using Tukey’s test at 0.05 level of significance.

The specimens were then removed from the device and the abutments unscrewed, and the parts of the interface between the rotation-prevention structure of the implants and abutments were examined using a scanning electron microscope (SEM).

## Results

**Fig. 2:**
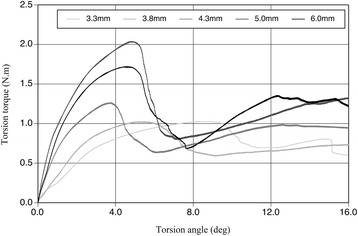
Torsional yield strength and maximum fracture torque strength of CAMLOG implant in each diameter

**Fig. 3:**
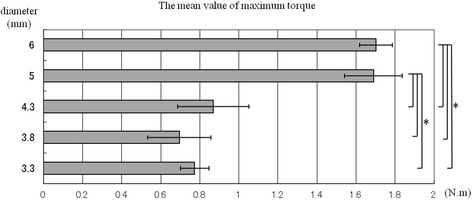
The mean value of maximum fracture torque strength

**Fig. 4:**
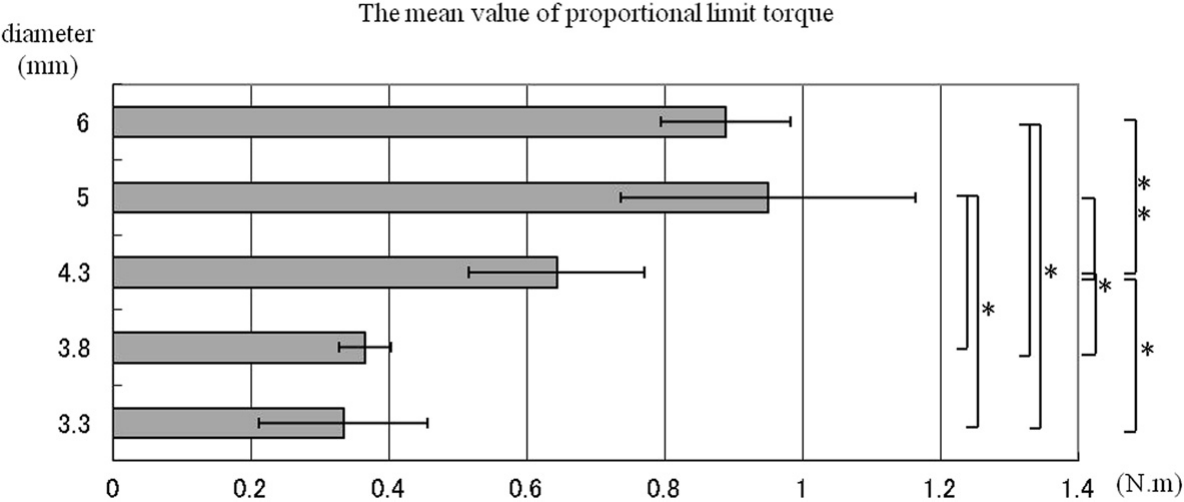
The mean value of torsional yield strength

**Fig. 5:**
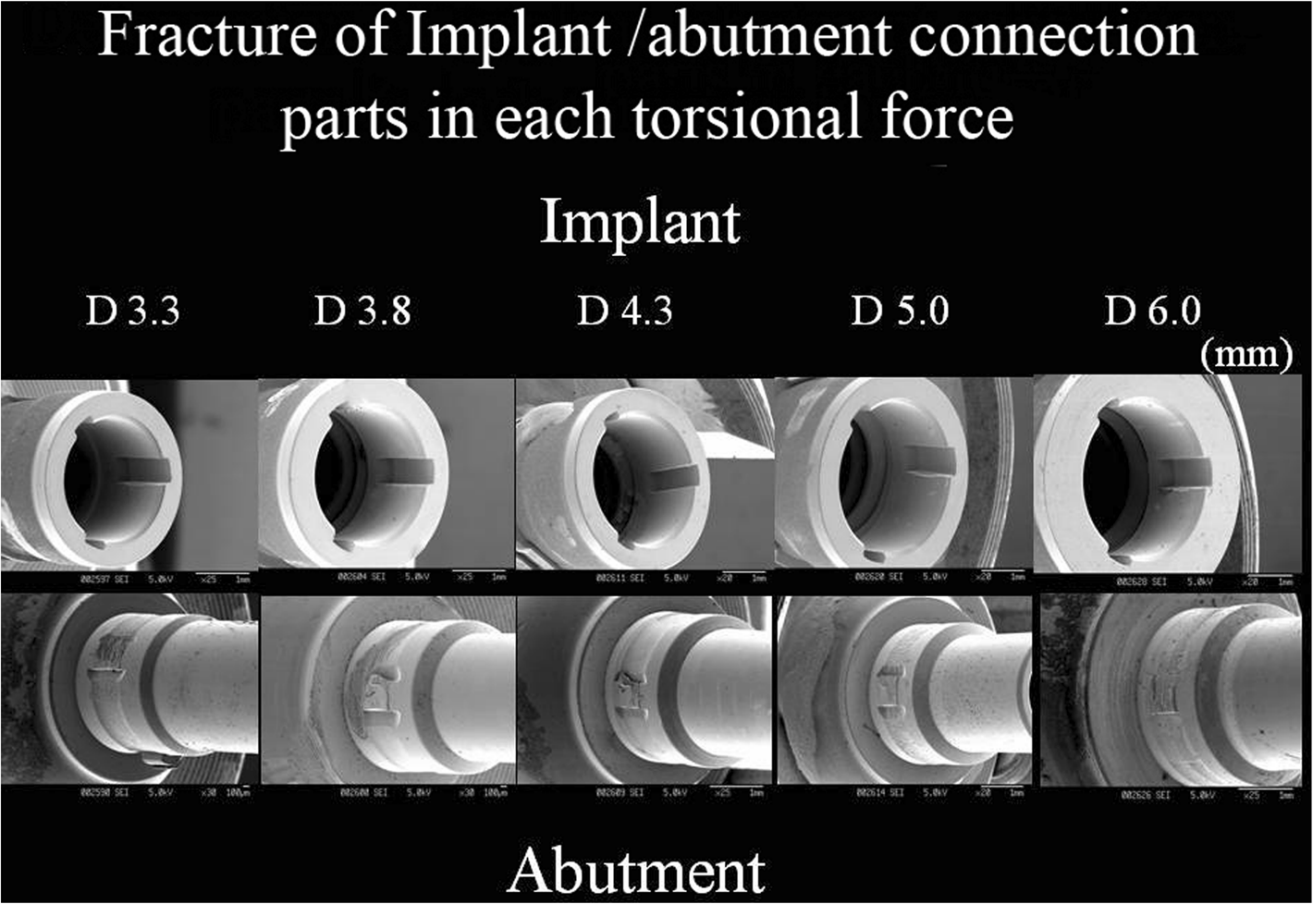
SEM picture of CAMLOG implant after torsion test

The relationship between torsional load and fracture occurrence followed a parabolic curve, and the maximum fracture torque and torsional yield strength of all sizes of implants were recorded (Fig. 2). The straight line of a primary curve in the initial phase represents the proportional limit. The transition point between the proportional straight line and the beginning of the curve is defined as the yield point and always occurs after plastic deformation. The mean values of these measured data are shown in Figs. 3 and 4. No significant differences in mean maximum fracture torque were found among 3.3, 3.8, and 4.3 mm-diameter implants, but significant differences were found between these sizes and 5.0 and 6.0 mm-diameter implants (*p* < 0.01). The mean torsional yield strengths demonstrated significant differences between 3.3, 3.8, and 4.3 mm-diameter and 5.0- and 6.0-mm-diameter implants (*p* < 0.01). The torsional fracture strength of the smaller diameter (3.3 and 3.8 mm) implants was markedly lower than that of the wider diameter implants. Observations under the SEM showed that all implan t-abutment connections had been damaged. Specifically, the internal notches of the implant body remained intact, but all projections of abutment corresponding to internal notches had been destroyed (Fig. 5).

## Discussion

During the physiological function of chewing, or the non-physiological function of bruxism, compressive, bending, and torsional stresses are generated in teeth or prosthetics. These stress will cause the abutment screw loosening or fracture, fracture of the abutment, fracture of the implant, and the implant/abutment connection. The extent of the damage is influenced by the design of the prosthesis, the fit of the implant prosthesis, the implant inclination, and the loading force. In these circumstances, tolerable enough clinical implant/abutment joint strength is required and ISO 14801 is used as their fatigue strength test method. However, torsional stresses generated in the oral cavity should also be considered. For these reasons, this study was undertaken in order to compare the torsional strength required to deform the implant-abutment connection for various diameter implants. The mode of failure will be the future investigation to be resolved by observing the fractured surfaces. In all specimens tested in this study, the relationship between static torsional load and fracture occurrence followed a parabolic curve. The primary curve was determined to be the torsional torque under which plastic deformation occurred and subsequently proceeded, resulting in permanent deformation. The curves illustrated in Fig. 2 showed two patterns in all specimens. Smaller diameter (3.3 and 3.8 mm) implants fractured much easier and earlier at the implant-abutment interface. This load-displacement curve is similar to the result of statistic loading test that Huang HM et al. [5] reported.

Examining all SEM images of the implant-abutment connections, we found that although their anti-rotational notches had been destroyed, the corresponding internal grooves remained intact. When torsion was applied, the grooves, which are composed of grade 4 commercially pure titanium (CP-Ti), were compressed; however, the abutment interlocks, which are composed of a titanium alloy (Ti-6Al-4V), were completely sheared off. Although the tensile strength of the Ti-6Al-4V alloy is at least 2 % greater than that of CP-Ti, much more titanium is adjacent to the grooves, which are compressed and can therefore withstand the transmitted force. On the other hand, the interlocks contain less material and therefore shear off if the applied force is too high. Nagel et al. studied the implant-abutment connection of each of the Replace-Select and the CAMLOG implants using FEM and reported that each design was very similar [15]. When comparing each system, they reported that the Replace-Select implant may fail by fracture of the implant body at the thinnest part of the wall. This thin portion represents the internal design that prevents the implant-abutment from rotating.

**Fig. 6:**
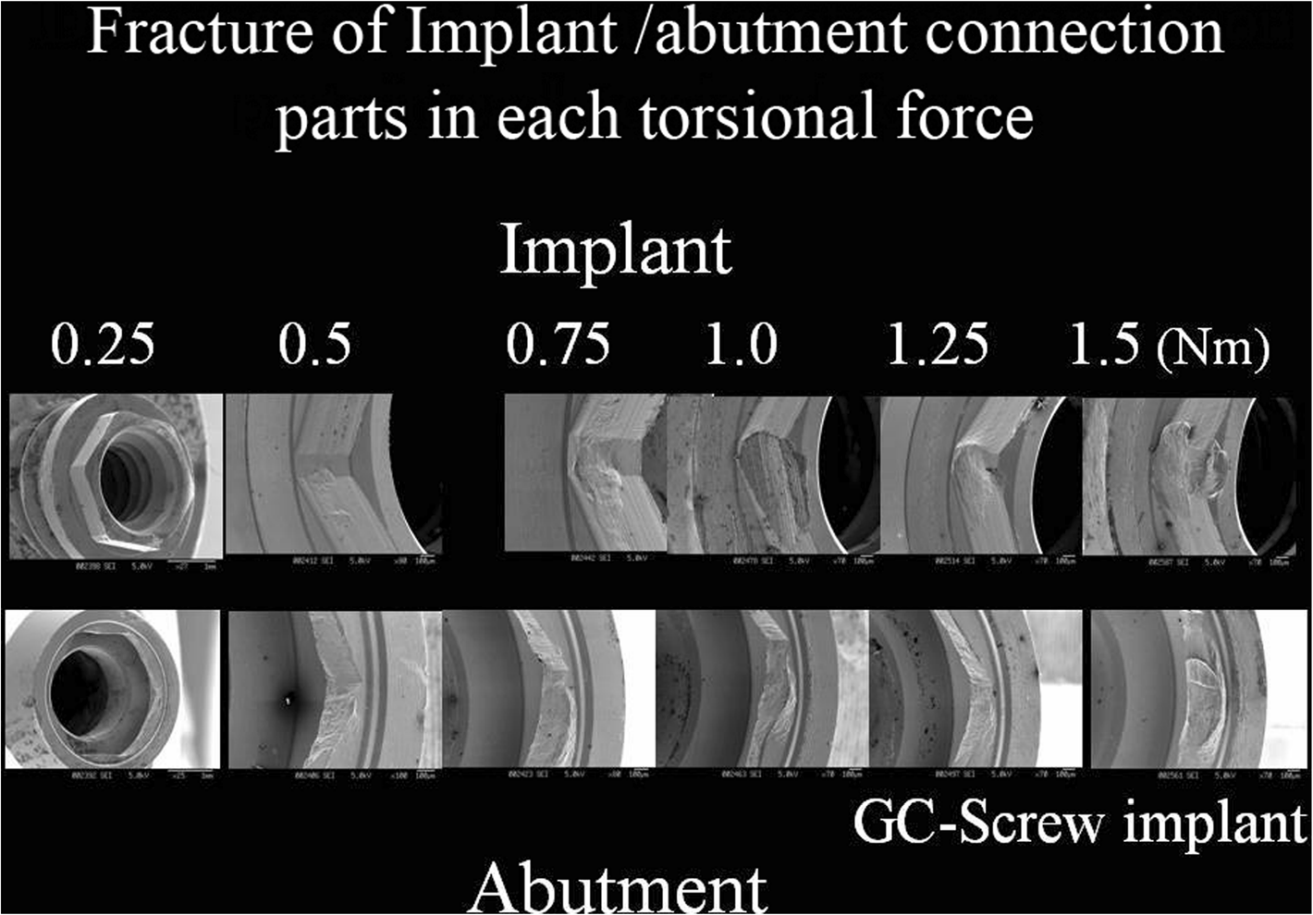
SEM picture of implant with external hexagonal connection

For comparison between the mechanical strength of CP-Ti screw implants with internal tube-in-tube implant-abutment connections and that of external hexagonal-type connections, all abutments and CP-Ti implants with external hexagonal-type connections were heavily damaged or destroyed in all phases of loading. A typical fracture curve for CP-Ti implants with external connections is shown. The proportional limit and a parabola-like curve with eternal destruction were drawn. Torsion forces of 0.25, 0.50, 0.75, 1.00, 1.25, and 1.50 N · m were applied to the external CP-Ti implants. Deformation occurred in both the implant and the abutment at each torsion force (Fig. 6). This might have been the result of the abutment connection design or the physical properties of the implant materials. In addition, the deformation effect on the torsional yield strength of the implants and abutments is worth noting, as deformation occurred immediately before torsion fracture in all specimens.

Balfour and O’Brien tested the following three kinds of implants for maximum anti-rotational stability: external hexagon-type 0.7 mm-diameter CP-Ti implants, internal octagon-type 0.6 mm-diameter Ti-6Al-4V implants, and internal hexagon-type 1.7 mm-diameter Ti-6Al-4V implants and abutments [16]. Testing comprised rigidly fixing a calibrated torque gauge to the abutment sleeve and applying torque until failure of the components was apparent. The torques necessary to separate the single-tooth abutments from the implants were 8.7 in.-lb (98.3 N · cm) for the external hexagon-type, 3.3 in.-lb (37.3 N · cm) for the internal octagon-type, and 10.0 in.-lb (192.1 N · cm) for the internal hexagon-type. In the internal octagon and internal hexagon designs, failure was limited to the abutment connections. The Balfour and O’Brien result differed from those reported in this study (4.3 and 3.8 mm diameters, 87 and 70 N · cm, respectively). The results from this study confirmed that the torsional strengths were different depending on the connection dimensions as reported by Balfour and O’Brien. CAMLOG implants (5 and 6 mm diameter) achieved higher torsional strength than 4.3, 3.8, and 3.3 mm diameter. This resulted from a combination of increased implant diameter and thickness of the implant walls.

## Conclusions

The forces that led to permanent deformation of the abutment connections were dependent on the implant diameter. The differences between the implants used in this study were obvious, both macroscopically and microscopically. Significantly less force was needed to fracture smaller than larger diameter implants. Furthermore, in the CAMLOG implants only, abutment projections corresponding to anti-rotational notches were destroyed, which could have been due to differences in the strength and tensile properties of the materials.
